# Surface Engineering of *Escherichia coli*–Derived OMVs as Promising Nano-Carriers to Target EGFR-Overexpressing Breast Cancer Cells

**DOI:** 10.3389/fphar.2021.719289

**Published:** 2021-11-18

**Authors:** Zahra Sepahdar, Mehran Miroliaei, Saeid Bouzari, Vahid Khalaj, Mona Salimi

**Affiliations:** ^1^ Department of Cell and Molecular Biology and Microbiology, Faculty of Biological Science and Technology, University of Isfahan, Isfahan, Iran; ^2^ Department of Molecular Biology, Pasteur Institute of Iran, Tehran, Iran; ^3^ Medical Biotechnology Department, Biotechnology Research Center, Pasteur Institute of Iran, Tehran, Iran; ^4^ Physiology and Pharmacology Department, Pasteur Institute of Iran, Tehran, Iran

**Keywords:** outer membrane vesicles, epidermal growth factor receptor, breast cancer, engineering, affibody

## Abstract

Bacterial outer membrane vesicles (OMVs) have recently drawn a great deal of attention due to their therapeutic efficiency and ability to target specific cells. In the present study, we sought to probe engineered OMVs as novel and promising carriers to target breast cancer cells. Following the fusion of the affi_EGFR_-GALA structure to the C-terminal of ClyA as an anchor protein, the ClyA-affi_EGFR_-GALA construct was successfully expressed on the surface of ∆msbB/∆pagP *E. coli* W3110-derived OMVs. Morphological features of the engineered and wild-type OMVs were identical. The engineered OMVs induced no endotoxicity, cytotoxicity, or immunogenicity, indicating the safety of their application. These OMVs could specifically bind to EGF receptors of MDA-MB-468 cells expressing high levels of EGFR and not to those with low levels of EGFR (HEK293T cells). Interestingly, despite a lower binding affinity of the engineered OMVs relative to the positive control Cetuximab, it was strong enough to identify these cells. Moreover, confocal microscopy revealed no uptake of the modified OMVs by the EGFR-overexpressing cells in the presence of EGFR competitors. These results suggest that OMVs might internalize into the cells with EGF receptors, as no OMVs entered the cells with any EGFR expression or those pretreated with EGF or Cetuximab. Regarding the EGFR-binding affinity of the engineered OMVs and their cellular uptake, they are presented here as a potential carrier for cell-specific drug delivery to treat a wide variety of cancer cells. Interestingly, the engineered OMVs are capable of reaching the cytoplasm while escaping the endosome due to the incorporation of a fusogenic GALA peptide in the construct.

## Introduction

Outer membrane vesicles (OMVs) are a kind of proteoliposomes that exist by nature with a diameter of 20–250 nm and are shed by Gram-negative bacteria through budding of the outer membrane (Bitto and Kaparakis-Liaskos, 2017). The OMVs mainly consist of the bacterial outer membrane molecules and enclose some periplasmic components (Nokleby et al., 2007; Ellis et al., 2010; Huang et al., 2016; Pathirana and Kaparakis-Liaskos, 2016; Vanaja et al., 2016). OMVs have been established as nanoparticle carriers that can be uptaken by the cells *via* ligand-dependent surface receptors (Furuta et al., 2009; Parker et al., 2010; Olofsson et al., 2014). Furthermore, the capability of engineering OMVs for targeted delivery of chemotherapeutic agents to certain cancer cell types makes them an appealing option for cancer treatment (Chen et al., 2010). It is worth noting that effective cancer chemotherapy usually requires a high-dose administration of drugs as these chemo drugs are prone to rapid clearance and poor circulating half-life (Iyer et al., 2013). This can result in severe and long-lasting side effects (Miller et al., 2016). In this sense, OMVs, as the naturally occurring nanoparticle delivery scaffolds, could be expected to serve as a promising drug delivery vehicle in cancer therapy.

A wide range of epithelial tumors, including breast cancer, is known to overexpress a transmembrane protein belonging to the ErbB receptor kinase family, namely EGFR (epidermal growth factor receptor 1) (Bhargava et al., 2005; Martinelli et al., 2009; Seshacharyulu et al., 2012; Changavi et al., 2015). In this regard, triple-negative breast cancer (TNBC), the most clinically aggressive subtype of breast cancer, is also associated with EGFR overexpression. The level of EGFR expression or gene mutation status seems to be important in clinical therapy, and it is being used to select patients for a specific treatment (Masuda et al., 2012). In this context, innovative anti-EGFR therapies have been developed in the last few years, including both monoclonal antibodies and small-molecule tyrosine kinase inhibitors (Flynn et al., 2009).

Taken together, engineered OMVs targeting EGFR in triple-negative breast cancer cells may provide a potential lead to specific antitumor therapy with low toxicity. To construct Affi_EGFR_-OMVs, the need for caution in selecting an appropriate anchor protein is vital to ensure the effective incorporation of a target recombinant protein structure onto the surface of membrane vesicles (MVs) without disrupting the vesicles or even the growth of the parent bacterium. Among the anchor proteins in *E. coli*, ClyA is a 34 KDa pore-forming cytotoxin with the ability to direct the desired protein to the surface of bacteria. This can be achieved by fusing the target sequence to the C-terminus of ClyA (Kim et al., 2008; Chen et al., 2010; Gujrati et al., 2014; Alves et al., 2015; Huang et al., 2016). Since ClyA is secreted on the surface of MVs of *E. coli*, it can thus provide an ideal platform to anchor recombinant proteins to the outer membrane (Kim et al., 2008). However, the detoxification of these OMV nanoparticles would be of great value due to enrichment of the outer membrane by LPS (Gu and Tsai, 1991). To solve this problem, LPS toxicity is partly attenuated by producing the LPS-modified OMVs harboring the strictly penta-acylated LPS from ∆msbB/∆pagP mutant of non-pathogenic *E. coli* W3110 by genetic modification (Lee et al., 2011).

In the present work, we employed bioengineered OMVs displaying an anti-EGFR affibody on their surface toward triple-negative breast cancer cells and sought to explore whether the engineered OMVs can represent a novel, safe, and targeted biological nanoparticle against triple-negative breast cancer as one of the most challenging types of breast cancer in terms of chemotherapy strategy. To expand the potential of our modified OMVs, we also utilized a fusogenic and pH-responsive amphipathic peptide, GALA peptide (Kakudo et al., 2004; Nishimura et al., 2014), thereby enabling the OMVs to escape the endosome. Functional assays were performed to assess the target specificity of the engineered OMVs toward the EGFR-positive and EGFR-negative cells. Furthermore, safety of the engineered OMVs was examined to validate their application as a novel cancer-targeting nanocarrier.

## Materials and Methods

### Bacterial Strains, Cell Lines, and Reagents

The *E. coli* strains used in this study included Top 10F’ as a host for plasmid propagations and cloning procedures, ∆msbB/∆pagP W3110, and BL21 (DE3), which were used to produce the engineered OMVs and recombinant protein, respectively. The mutant host strain was kindly provided by Prof. Sang Hyun Kim (KRIBB, Republic of Korea) and the remaining two along with the protein expression vectors pET-32a (+) and pET-28a (+) were purchased from Invitrogen (Carlsbad, CA, United States). *E. coli* strains were grown in Luria–Bertani (LB) medium [1% (w/v) tryptone, 0.5% (w/v) yeast extract, and 1% (w/v) NaCl, pH 7.0]. The growth medium was supplemented with ampicillin (100 µg/ml) and kanamycin (50 µg/ml) when required. Restriction endonucleases and T4 DNA ligase were supplied by Fermentas (Waltham, United States) and Roche companies (Penzberg, Germany).

MDA-MB-468 (human breast carcinoma), HEK293T (human embryonic kidney), and THP-1 (human monocytic) cell lines were obtained from the cell bank of Pasture Institute of Iran (NCBI). Cells were cultured in Dulbecco’s Modified Eagle’s Medium (DMEM) or RPMI1640 medium containing 10% fetal bovine serum (FBS), 100 U/mL penicillin, and 100 μg/ml streptomycin (GibcoBRL, Rockville, IN, United States) at 37°C with 5% CO_2_ in a humidified atmosphere inside a CO_2_ incubator. All reagents used were of analytical grade and obtained from Merck (Darmstadt, Germany) and Sigma-Aldrich (MO, United States). EGF antigen was a kind gift from Dr. Majid Golkar (Pasteur Institute of Iran, Tehran, Iran).

### Preparation of ClyA-Affibody-GALA Expression Cassette

A codon-optimized 1,356 bp DNA fragment encoding His tag (Gly_4_Ser)_3_, myc tag, Z_EGFR_:1907 affibody (Gly_4_Ser)_3_, and GALA fused to the C-terminus of Cytolysin-A gene was synthesized by GeneRay Biotech (Shanghai, China) and delivered in an intermediate pMD18-T plasmid. To facilitate the expression procedure in *E. coli* W3110, we first generated a modified version of pET32a (mpET32a) in which the T7 promoter was replaced by Tac promoter. This step was performed to bypass the T7 RNA polymerase-dependent expression of our construct in *E. coli* W3110. Next, the ClyA-affibody-GALA construct was cloned in the HindIII/NcoI site of mpET32a. Finally, the recombinant plasmid, mpET32a/ClyA-affibody-GALA, was transformed into the ∆msbB/∆pagP W3110 *E. coli* strain and the transformed strain was maintained on the solid medium supplemented with ampicillin (100 μg/ml).

### Preparation of the Affi_EGFR_-OMVs

OMVs were isolated according to the procedure described previously by Chen et al. (2010) with some modifications. Briefly, the overnight saturated cultures of *E. coli* were inoculated into 1 L of LB medium supplemented with ampicillin (1:100 v:v). Subculturing was carried out in the baffled flask using a 1:5 (v:v) ratio of liquid to air to enhance the yield of vesicle production. The culture was shaken at 30°C for 5 h until cells reached the mid-log phase (OD600 ∼ 0.6–0.8). Then, the required amount of isopropyl β-d-1-thiogalactopyranoside (IPTG) was added to a final concentration of 1 mM and the cultivation was continued for a further 20 h at 20°C to induce expression of the recombinant protein. Afterward, the bacterial pellet was precipitated by centrifugation at 5,000 × g (20 min) and cell-free culture supernatant was collected and filtered through a 0.45 μm filter and concentrated to 80 ml by ultrafiltration through an Amicon filter with 100 kDa cutoff (Millipore, Billerica, MA, United States). Lastly, OMVs were isolated by ultracentrifugation (Ti98 rotor, Beckman-Coulter, California, United States) at 170,000 × g for 2 h at 4°C. The purified OMVs were washed with phosphate-buffered saline (PBS) at 170,000× g for an additional 2 h, followed by resuspending the pellet in 1 ml of PBS and passing through Detoxi-Gel endotoxin-removing columns (Pierce, Thermo Scientific, United States) and 0.20 μm cellulose acetate filters. The purified OMVs were stored at −20°C until use. The total protein concentration of OMVs was quantified using Bradford assay, and the bovine serum albumin was used as a standard.

### Preparation of Recombinant Affi_EGFR_-OMVs

The gene fragment encoding His tag, G4S, myc tag, Z_EGFR:_1907 affibody, G4S, and GALA peptide was PCR-amplified using affibody forward (5-GAG​GTA​CCG​GAA​GTC​GGG​GGC​GG -3′) and affibody reverse (5′-CTC​TAG​ATT​ATT​TAG​GAG​CCT​GTG​CAT​C-3′) primers using pMD18-T vector as a template. The amplified sequence was then cloned between KpnI and XbaI sites of the pET28a expression vector and the final construct was transformed and expressed in *E. coli* BL21 (DE3) strain. To express the recombinant protein, a 500 ml of bacterial culture supplemented with kanamycin was set and the protein expression was induced by adding IPTG (1 mM) at 37°C for 4 h. The bacterial pellet was then collected and lysed using lysis buffer (300 mM NaCl, 50 mM NaH_2_PO_4_, and 10 mM imidazole; pH8.0) and sonication (12 cycles of 20 s ON/OFF). Following centrifugation at 5,000 × g for 20 min, the supernatant was loaded on the Ni-NTA agarose column (QIAGEN; Hilden, Germany) equilibrated with the same lysis buffer. After washing, the recombinant protein was eluted using the elution buffer (300 mM NaCl, 50 mM NaH_2_PO_4_, and 250 mM imidazole; pH8.0) and finally, imidazole was removed from the protein solution and replaced with PBS using an Amicon ultrafiltration system (3 kDa cutoff filter) (Millipore, Billerica, MA, United States). The concentration of the purified fusion protein was determined by the Bradford assay.

### Characterization of the Affi_EGFR_-OMVs

Expression of the exogenous protein was detected by electrophoresis. Briefly, bacterial cells and OMV samples were suspended in lysis buffer (4% CHAPS, 7M Urea, 2M Thiourea, and 40 mM Tris-HCl) and boiled for 15 min in a loading buffer containing β-mercaptoethanol. After cooling to room temperature, samples were loaded onto a 10% polyacrylamide gel and separated electrophoretically. To visualize protein bands, SDS-PAGE gels were stained with Coomassie Brilliant Blue R-250 (BioRad, United States).

For Western blot analysis, bacterial cell lysates and OMV samples were separated by SDS-PAGE as described above and the protein bands were subsequently transferred to a PVDF membrane *via* semidry transfer. Membranes were blocked using skim milk in PBS (3% w/v) and the fusion protein band was detected by a mouse horseradish peroxidase (HRP)-labeled monoclonal antibody against the 6XHis-tag (1:1,000 v/v, Sigma-Aldrich, United States, A7058). For further verification, specific antibodies, including goat polyclonal anti-affibody primary antibody (1/4,000 v/v, Abcam, United Kingdom, ab50345) and HRP-conjugated anti-goat secondary antibody (Abcam, United Kingdom), were used. Bands were visualized using HRP substrate Clarity ECL kit (GE Health Care Life Sciences, Buckinghamshire, United Kingdom).

To confirm the surface distribution of ClyA-affibody-GALA in bacteria and OMVs, immune staining of the samples was performed and the samples were analyzed using flow cytometry. In brief, bacterial cells were centrifuged at 8,000 × g for 5 min and then washed with PBS at 8,000 ×g for another 5 min. Afterward, bacteria were blocked in PBS containing 3% (w/v) BSA for 15 min and incubated with anti-affibody antibody in 1% (w/v) BSA (1:500) for 1 h. Following washing, samples were incubated with FITC -conjugated secondary antibody (1:2000) (Abcam, United Kingdom) for 30 min. Flow cytometry analysis was performed using the CyFlow® SL machine (Partec, Munster, Germany). For OMVs, at the end of each step, OMVs were collected using a 50 kDa cutoff ultrafiltration tube (Millipore, Billerica, MA, United States) and centrifugation at 5,000 × g for 30 min (Huang et al., 2016).

### Determining the Size and Morphology of the Affi_EGFR_-OMVs

Transmission electron microscope (TEM) analysis was performed for structural analysis of vesicles. For this purpose, fresh non-targeted and EGFR-targeted OMVs (modified OMVs, mOMVs) (250 µg/ml) were resuspended in 3% glutaraldehyde with the ratio of 1:1 to fix them. Next, one drop of this solution was mounted on a Formvar coated grid. Samples were then negatively stained with 2% uranyl acetate for 2 min and air-dried for 1 hour. Upon washing with distilled water, the completely dried copper grids were examined under a transmission electron microscope Zeiss EM900, 80 kV (Germany), to assess the size and morphology of OMVs.

### Analysis of Size Distribution of OMVs

Size distribution of non-targeted OMVs and the affi_EGFR_-OMVs was measured by dynamic light scattering (DLS) using a ZetaSizer Analyzer (Malvern Instruments Ltd. Nano-ZS ZEN3600, United Kingdom) equipped with a 5 mW HeNe laser and operating at an angle of 173°. To detect Z-average size and polydispersity index (PDI) of the OMVs, the OMV frozen samples were thawed at room temperature and diluted to 50 μg/ml (total protein concentration) in PBS. Z-average defines the mean diameter of the vesicles in nm (d.nm), while PDI describes the particle size distribution. Tracing analysis was performed in three independent runs for each sample in single-use polystyrene zeta cell DTS1060 with a path length of 10 mm by measuring the backscattering intensity at 25°C. Scattering light detected at 173° was automatically adjusted by laser attenuation filters. The software used to collect and analyze the data was the Zetasizer software version 7.01. Size distribution by intensity was preferred to measurements by number or by volume to have more reproducible results.

Furthermore, the alteration in size and PDI of affi_EGFR_-OMVs reflecting vesicles stability was tested during storage for a period of 3 days at 4°C.

### Tyndall Effect

The Tyndall effect was applied to verify the existence of OMVs as nanoparticles in the solution using a red laser pointer as the light source. A visible light beam path can be observed in the nanoparticle solution due to Tyndall scattering.

### Monitoring the Specific Binding of the Affi_EGFR_-OMVs Using Enzyme-Linked Immunosorbent Assay

Enzyme-linked immunosorbent assay (ELISA) was carried out to explore the specificity of the engineered OMVs for EGFR receptors according to a previously described protocol with some modifications (Kim et al., 2012). Briefly, 100 μL of EGFR protein (5 µg/ml) (Abcam, United Kingdom) and bovine serum albumin (BSA) (5 μg/ml in PBS) were coated onto 96-well plates (SPL, Korea) and incubated at 4°C overnight. Following removal of the coating solution, 200 μL of blocking buffer containing 2% of BSA was added and further incubated for 2 h at 25°. Afterward, the plates were washed four times with PBS containing 0.1% of Tween-20 and exposed to 100 μL of the OMVs or affi_EGFR_-OMVs at different concentrations (5–150 μg/ml) and incubated for 2 h at 25°C. The affi_EGFR_-OMVs were then detected using 100 μL of goat polyclonal anti-affibody primary antibody (1/4,000 in PBST), followed by four times washing and incubating with horseradish peroxidase (HRP)-conjugated secondary antibody. The immunoreactivity was observed using the chromogenic HRP substrate 3,3′,5,5′-tetramethybenzidine (TMB) (Abcam, United Kingdom) and the absorbance was measured at 450 nm. Finally, the function of the engineered OMVs was compared with the affi_EGFR_ protein (0.1–10 μg/ml).

ELISA was also performed with cancer cell lines. MDA-MB-468 (EGFR-positive) and HEK293T (EGFR-negative) cell lines were grown in a DMEM medium containing 10% FBS. Cells (10^6^/ml) were lysed by sonication (15 cycles: 10 s on/10 s off) at 80 A and then 100 μL of the cell lysate was coated onto each well (Banisadr et al., 2018). Upon blocking and washing, coated wells were incubated with 100 μL of the affi_EGFR_-OMVs and OMVs at 55 μg/ml as well as affi_EGFR_ (3.5 μg/ml) and Cetuximab (Erbitox®, Merck) (1.5 μg/ml) as the positive control. HRP-conjugated anti-human secondary antibody (Cytomatin gene, Isfahan, Iran) was applied to detect Cetuximab binding.

### Cell Binding Assessment by Flow Cytometry

Cell specificity and binding capacity of the affi_EGFR_-OMVs were examined by flow cytometry as described before (Zarei et al., 2014). In brief, 5×10^5^ cells of each of MDA-MB-468 and HEK293T were spun down at 800×g for 5 min. Cells were resuspended in 100 μL of FACS buffer (PBS, 5% FBS) and incubated with 25 μg of OMVs and affi_EGFR_-OMVs or 1.5 μg of the affi_EGFR_ for 2 h at 4°C. Following washing two times with FACS buffer and blocking, cells were incubated with 100 μL of anti-affibody antibody (1/500) for a further 2 h at 4°C, then stained with FITC -conjugated secondary antibody (1/2000) in the dark for 30 min at 4°C, and finally resuspended in PBS. The fluorescent intensity of the cells was measured by flow cytometry. All samples were compared to negative control. For the negative control, the cells were processed and stained in the same manner with anti-affibody and FITC-conjugated secondary antibody, except for incubating with OMVs or affi_EGFR_. To quantify the difference in cell binding, the mean fluorescence intensity was calculated for all the treatments.

### Competitive Binding Assay by Flow Cytometry

The specific binding of the modified OMVs was more investigated in the presence of the EGFR competitors. For this goal, 5×10^5^ cells of EGFR-positive MDA-MB-468 cells were pretreated with an excess amount of EGF (7.5 μg) or Cetuximab (186.5 μg) in 200 μL of FACS buffer (equal to 12.5 μM) for 90 min at 4°C. Having washed the cells, they were then treated with 25 μg of OMVs or affi_EGFR_-OMVs for a further 90 min at 4°C. Anti-affibody antibody and FITC-conjugated secondary antibody were applied as previously described. The fluorescent intensity of the cells was measured by flow cytometry.

The binding specificity of the affi_EGFR_-OMVs was also examined with the increasing concentrations of EGF as an EGFR competitor. To do it, MDA-MB-468 cells (5×10^5^ cells/200 μL per tube) were incubated with 25 μg of affi_EGFR_-OMVs in the presence of 15 ng to 7.5 μg (3.25 nM–12.5 μM final concentration) of EGF for 2 h at 4°C. Anti-affibody and FITC were used for staining cell-bound affi_EGFR_-OMVs. MFI was recorded and % of cell binding of affi_EGFR_-OMVs + EGF tests was calculated relative to affi_EGFR_-OMVs alone.

### Evaluation of EGF Receptors Expression and Phosphorylation by Western Blotting Analysis

To detect expression of EGFR in MDA-MB-468 and HEK293T cells, the cells were seeded at 2.5×10^5^ in 6 well plates and incubated at the standard growth culture for 24 h. For the EGFR stimulation test, MDA-MB-468 cells were plated and treated with affi_EGFR_-OMVs (250 μg/ml) or EGF (100 nM) on the next day following overnight serum starvation. The cells were then lysed using RIPA lysis buffer (Sigma-Aldrich) containing protease inhibitors. The protein concentration was determined using the Bradford protein assay and protein samples were separated by 10% SDS-PAGE electrophoresis. Western blotting analysis was then performed as described before. Images were quantified using the ImageJ 1.4.3.67 (NIH software). The protein content was normalized to the level of β-actin. The antibodies used were as follows: EGFR (1/1,000, orb225296, Biorbyt, United Kingdom), p-EGFR (1/200, SC-81488, Santa Cruz Biotechnology, United States) and β-actin (1/1,000, 4967S, Cell Signaling, United States).

### Monitoring the Cell Binding and Cell Uptake of Affi_EGFR_-OMVs by Confocal Microscopy

To visualize the cell binding of OMVs, confocal microscopy was applied (Gujrati et al., 2014). In a few words, 1.5 ×10^5^ of MDA-MB-468 and HEK293T cells were seeded on 8-well glass slides (SPL Life Sciences, Korea) in a DMEM medium containing 10% FBS and antibiotics for 24 h to reach 60% of confluency. Cells were then fixed with paraformaldehyde (PFA, 3.7% in PBS) for 15 min, blocked by 2% of BSA for 1 h at room temperature, and then further incubated with the affi_EGFR_-OMVs (25 μg) for 2 h at 4°C. Samples were sequentially exposed to anti-affibody primary antibody (1:500) and FITC-labeled secondary antibody (1:2,000). Cells were finally evaluated under a confocal microscope (Olympus Fluoview TM FV 1000, Olympus, Japan).

For tracing the cellular uptake of OMVs, MDA-MB-468 cells at 60% of confluency were exposed to the OMVs and affi_EGFR_ OMVs (25 μg) for 2 h at the standard growth culture. After being washed with PBS, cells were fixed in 3.7% of PFA at room temperature for 10 min. Fixed cells were then washed and permeabilized by 0.1% Triton X-100 for 10 min. Avoiding nonspecific interaction of antibodies, cells were treated with 3% (w/v) of bovine albumin serum (BSA) in PBS for 1 h at room temperature. Cells were finally incubated with anti-affibody primary antibody and FITC-labeled anti-goat secondary antibody. The nuclei were stained with propidium iodide (PI, 1 μg/ml) (Sigma-Aldrich, MO, United States) and cells images were taken by the confocal microscope.

To investigate the uptake of affi_EGFR_-OMVs by MDA-MB-468 cells, EGFR-overexpressing cells were initially incubated with EGF (7.5 μg) or Cetuximab (186.5 μg) for 2 h in a standard culture medium. Following washing, cells were exposed to 25 μg of OMVs or affi_EGFR_-OMVs and incubated for a further 2 h at 37°C. The procedure was similar to that described for tracing cell uptake. Finally, the treated cells were evaluated using a confocal microscope.

### Measuring the Affi_EGFR_-OMVs Using the Gel Clot Limulus Amoebocyte Lysate Assay

Endotoxicity of affi_EGFR_-OMVs in both the intact and lysed (with 0.5% of sodium deoxycholate) forms was evaluated using the commercial gel clot LAL kit (PYROTELL, Associates of Cape Cod Inc. United States). Serial dilutions of affi_EGFR_-OMVs and its lysate (250 down to 7.81 μg/ml) were applied in this test, and endotoxin activity was assayed as per the manufacturer’s instructions in duplicate. Control standard endotoxin (CSE) (*E. coli* strain O111:B4, 0.5 EU/mL) and LAL reagent water (LRW), provided by the Associates of Cape Cod Inc., were used as positive and negative controls, respectively.

### Sterility and Hemolytic Activity of the Affi_EGFR_-OMVs

To assess sterility of the affi_EGFR_-OMVs, OMV preparations were plated on LB-agar plates and grown overnight at 37°C. Cytolytic or hemolytic activity assay was performed using horse blood agar plates. To do this, the affi_EGFR_-OMVs-derived (500 μg/ml) and its parent strains (transformed ∆msbB/∆pagP W3110) were plated on the horse blood agar plates and incubated at 37°C overnight. β-*Streptococcus* was also plated as a positive control. The next day, plates were analyzed for any clear zone as a result of red blood cells lysis.

A liquid hemolysis assay was also performed to show the hemolysis activity quantitatively. To this end, human erythrocytes were washed and diluted with a ratio of 2:100 in PBS. Aliquots of the washed erythrocytes were then transferred to a v-bottom 96-well plate and subjected to affi_EGFR_-OMVs (6.25–400 μg/ml) for 1 h at 37°C. Upon centrifugal sedimentation of the cells and debris at 2000 rpm for 5 min, the supernatant hemoglobin was quantified by spectrophotometric detection at 540 nm. PBS and deionized water were used as negative and positive controls, respectively. Hemolysis activity was reported relative to the erythrocytes lysed in deionized water.

### Determining Cell Viability Following Outer Membrane Vesicles Exposure

Cell viability was measured following OMVs exposure *via* MTT assay in the MDA-MB-468 and HEK293T cell lines. In short, a total of 1 × 10^5^ cells were seeded in 96-well plates overnight and treated with both OMVs and the affi_EGFR_-OMVs (25–400 μg/ml) for 4 h. The medium was then refreshed and the cells were further incubated for 72 h in a standard growth condition. To confirm the nontoxic effect of OMVs, the percentage of cell viability was calculated. Cisplatin (Sigma-Aldrich, MO, United States) was also applied as a positive control.

### THP1 Cell Stimulation

The procedure for this assay was as described previously with certain modifications (Widdrington et al., 2018). Briefly, THP-1 cells were grown as a suspension culture in RPMI-1640 medium containing 10% FBS. Cells were then exposed to 200 nM of phorbol myristate acetate (PMA) for 48–72 h to induce differentiation. Afterward, the obtained adherent cells were treated with affi_EGFR_-OMVs (25–400 μg/ml) along with PBS and LPS (100 ng/ml) (Sigma-Aldrich, MO, United States) as negative and positive controls, respectively. Following a 24 h incubation, human TNF-α was quantified using ELISA (Quantikine HS, United States) as per the manufacturer’s instructions.

### Statistics

All statistical analyses were performed using GraphPad Prism 8 (GraphPad Software, La Jolla, CA, United States). Differences were considered significant if the *p* value was <0.05. All graphed values represent the mean, and the error bars show standard error. One-way analysis of variance (ANOVA) followed by *post hoc* Tukey’s test was used for intergroup comparisons.

## Results

### Recombinant ClyA-Affi_EGFR_-GALA Was Expressed on W3110 *E. coli*

**FIGURE 1:**
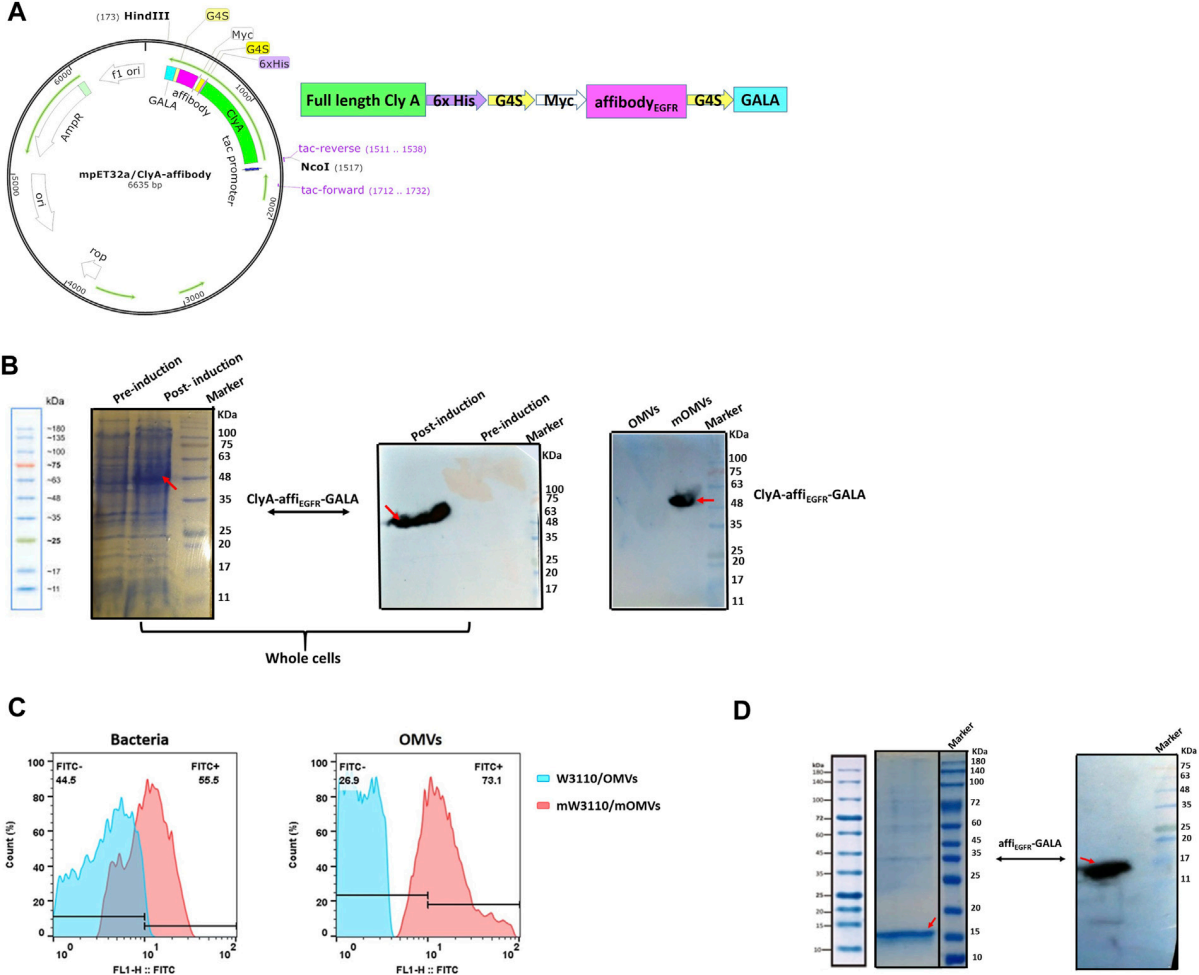
Protein expression analysis. **(A)** Schematic diagram of ClyA-affi_EGFR_-GALA construct. The orientation of fragments used in the construct has been shown. **(B)** Expression of the ClyA-affi_EGFR_-GALA fusion protein was evaluated in ∆msbB/∆pagP W3110 using 10% SDS-PAGE and Western blotting in total bacterial protein samples **(left and middle)** and the engineered OMVs **(right)** (mOMVs: modified OMVs). **(C)** Immune fluorescence analysis of the location of ClyA-affi_EGFR_-GALA. ∆msbB/∆pagP W3110 cells and their derived OMVs were surface stained using an anti-affibody antibody. **(D)** 12% SDS-PAGE **(left)** and Western blotting **(right)** of the purified affi_EGFR_-GALA in BL21 (DE3).

The ∆msbB/∆pagP W3110 *E. coli* K12 strain is a nonpathogenic strain harboring two mutations in msbB and pagP genes to highly reduce endotoxin activity (Lee et al., 2011). ClyA-affi_EGFR_-GALA construct (Figure 1A) was transformed into this strain to prepare the affi_EGFR_-OMVs with the least cytotoxic effect.

As illustrated by SDS-PAGE, a clear protein band of ∼50 kDa, corresponding to the full-length ClyA-affi_EGFR_-GALA along with tags, was detected in the whole-cell lysate of recombinant W3110, indicating that the fusion protein had efficiently been expressed in W3110 cells. Furthermore, the presence of the fusion protein in the OMVs derived from the engineered ∆msbB/∆pagP W3110 samples was confirmed by immunoblotting using anti-6XHis-tag (data not shown) and anti-affibody antibody (Figure 1B). Detection of a reacting band of about 50 kDa further confirmed the successful expression of the ClyA-affi_EGFR_-GALA fusion protein in the OMVs.

Next, to verify whether the fusion protein was present on the surface of OMVs, the intact W3110 cells and OMVs were subjected to anti-affibody antibody treatment and analyzed by flow cytometry. Our findings confirmed the presence of affi_EGFR_-GALA on the surface of OMVs. In other words, cells and OMVs displaying affi_EGFR_ were brightly fluorescent, while no fluorescence was detected for the untransformed cells and OMVs (Figure 1C). To validate these findings, we also expressed and then purified the affi_EGFR_ recombinant protein (16 KDa) as a positive control in BL21 DE3 (Figure 1D).

Taken together, using genetic engineering techniques, the affi_EGFR_-GALA was successfully presented on the surface of ∆msbB/∆pagP W3110-derived OMVs *via* fusion to ClyA.

### Characterization of the Engineered Outer Membrane Vesicles

#### Transmission Electron Microscopy

**FIGURE 2:**
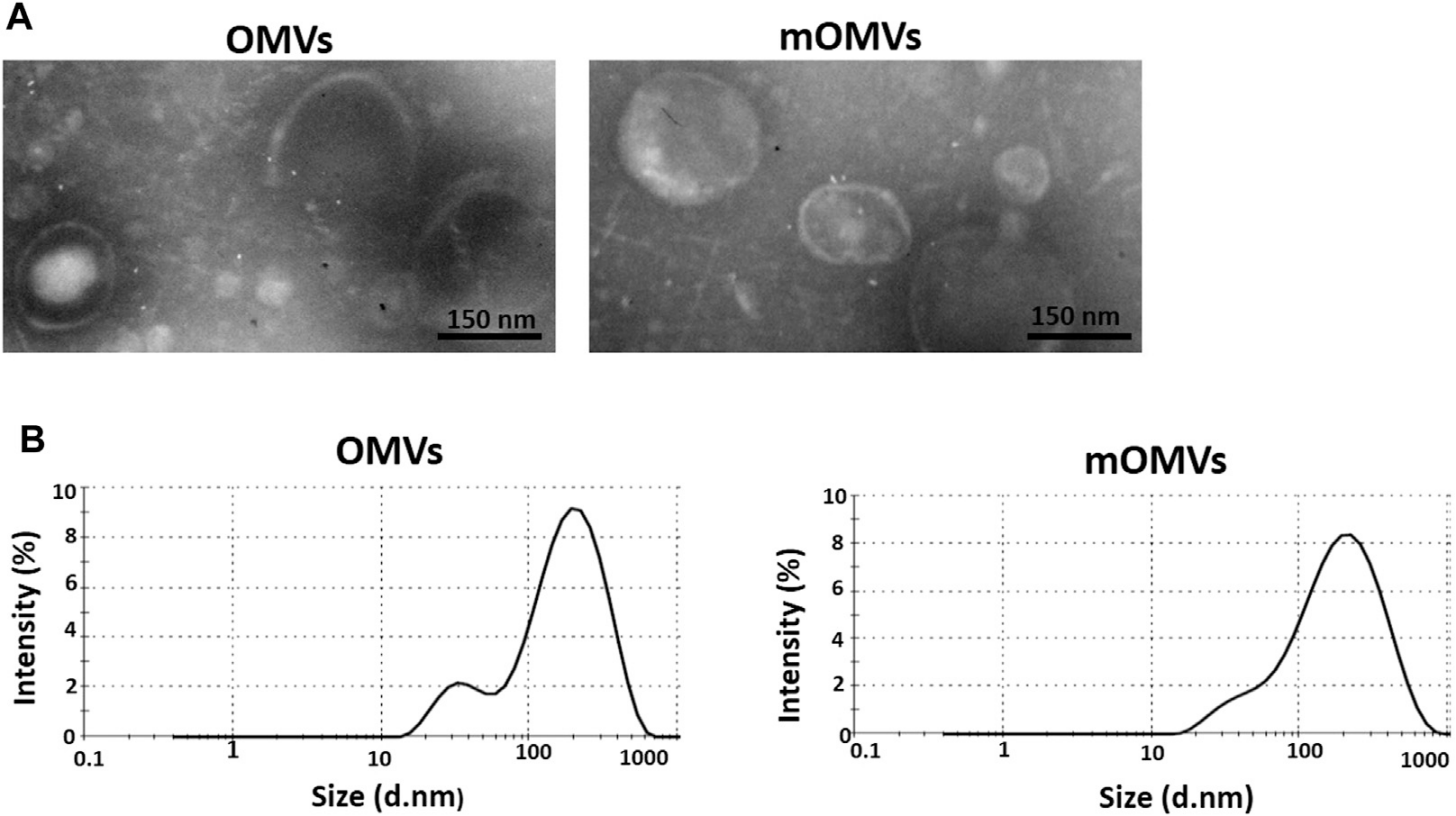
Characterizing the engineered OMVs. **(A)** Transmission electron microscopic observation of the OMVs **(left)** and modified OMVs (mOMVs) **(right).** The bar indicates 150 nm. **(B)** Dynamic light scattering confirmed the size distribution of OMVs.

Morphological and structural alterations of the engineered OMVs were examined by TEM. The findings exhibited spherical nanoscale vesicles, which in comparison to non-targeted OMVs, no significant difference in morphology was detected in the modified OMVs (affi_EGFR_-OMVs). These results suggest that the association of ClyA-affi_EGFR_-GALA in the engineered OMVs had no apparent effect on the size of the particles (Figure 2A).

### Dynamic Light Scattering and Tyndall Effect

The identical size distribution of the OMVs and affi_EGFR_-OMVs was confirmed by dynamic light scattering analysis (Figure 2B). As reported in Table 1, there was no significant difference (*p* > 0.05) in terms of polydispersity index (PDI), maximum peak (nm), and Z-average (d.nm) between two types of OMVs. These results were in parallel with the TEM findings confirming that incorporation of a heterologous protein did not alter the size of the affi_EGFR_-OMVs. To verify the stability of the OMVs, the frozen OMVs were thawed and the above parameters were determined on three consecutive days at 4°C. As indicated in Table 2, no significant alteration was observed in terms of PDI and Z-average.

**TABLE 1:** Size distribution parameters of OMVs and modified OMVs (mOMVs) determined by DLS^a^.

Maximum peak (nm)		Z-Average (d.nm)	PDI
OMVs	192.26 ± 14.55	136.26 ± 38.15	0.417 ± 0.044
mOMVs	186.36 ± 25.98	135.76 ± 30.33	0.370 ± 0.043

a Values were expressed as mean ± SD from three independent runs.

**TABLE 2:** Size and PDI value of vesicles of mOMVs stored at 4°C during 3 days.

	Day 1	Day 2	Day 3
PDI	0.369	0.372	0.426
Z-Average (d.nm)	167.4	228.2	238.9

Moreover, to demonstrate the colloidal feature of the OMVs solution, the Tyndall effect was evaluated in the samples. The Tyndall effect is the phenomenon indicating the divergence of a light beam when it passes through a colloidal dispersion and a portion of the light is scattered. As it can be seen from Supplementary Figure S1, the PBS buffer sample has no obvious Tyndall effect, while the OMVs solution sample has a strong Tyndall effect confirming the presence of OMVs.

### Affi_EGFR_-OMVs Specifically Bound to Epidermal Growth Factor Receptor-1 Protein in ELISA Test

**FIGURE 3:**
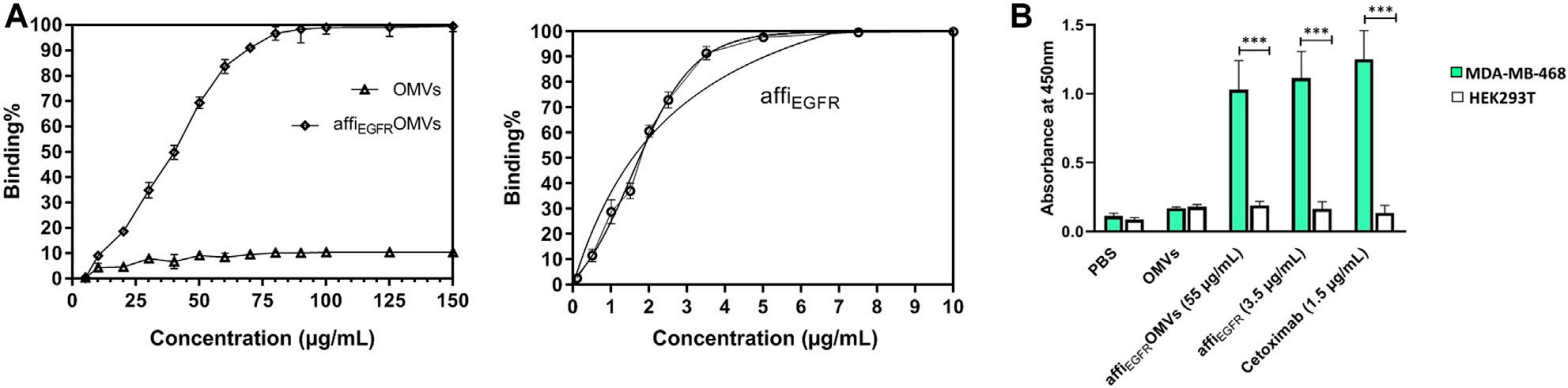
Binding capacity of the OMVs, affi_EGFR_-OMVs, and affi_EGFR_ to EGFR protein was assessed using ELISA. **(A)** Affi_EGFR_-OMVs and the purified affi_EGFR_ attach to EGFR protein, while no EGFR binding is observed for non-targeted OMVs even at the highest concentration. **(B)** Affi_EGFR_-OMVs and the purified affi_EGFR_ specifically bind to the MDA-MB-468 cells expressing EGFR. Cetuximab was applied as a positive control for EGFR-specific binding. OMVs and the purified protein were explored by consecutive incubation with anti-affibody antibody and HRP- conjugated rabbit anti-goat IgG. Data are expressed as the mean ± SEM from three independent experiments (***p < 0.001).

Affi_EGFR_-OMVs have to identify EGFR protein in ELISA. For further verification, affi_EGFR_ protein was used as a positive control. As shown in Figure 3A, both affi_EGFR_-OMVs and affi_EGFR_ interact with the EGF receptor in a concentration-dependent manner; i.e., the higher the affi_EGFR_-OMVs concentration, the greater the strength of binding. It is worth mentioning that the graph in Figure 3A finally reaches a saturation plateau. As expected, affi_EGFR_ was effective even at a low applied concentration. Interestingly, no absorbance was detected for the OMVs even at the highest applied concentrations (Figure 3A). Our calculated K_d_ values were 86.41 and 2.845 μg/ml for the engineered OMVs and affi_EGFR_, respectively. Basd on the K_d_ values, it is suggested that 1 μg of the affi_EGFR_-OMVs includes 32 ng of affi_EGFR_ corresponding to about 3% of the total protein.

### ELISA Assay of Cell Lysate Confirmed Affi_EGFR_-OMVs Specific Binding

For further validation, an ELISA test was also carried out on the cell lysates. Both affi_EGFR_-OMVs and affi_EGFR_ interacted with MDA-MB-468 cells. A high and reliable concentration (∼EC_90_) was used for satisfactory results. The results showed that the absorbance values for affi_EGFR_ and affi_EGFR_-OMVs interacting with MDA-MB-468 cell lysate were significantly higher than those for the HEK293T cell lysate. As expected, HEK293T cells did not interact with either affi_EGFR_-OMVs, affi_EGFR_, and Cetuximab owing to their lack of receptor (Figure 3B). These results are in line with our immunoblotting findings verifying no EGF receptor expression in HEK293T cells (Supplementary Figure S2). Moreover, no significant difference in terms of OD values was detected following incubation of both MDA-MB-468 and HEK293T cells with non-targeted OMVs. In contrast, Cetuximab as a positive control exhibited a high OD value at the concentration applied in MDA-MB-468 cells.

In summary, the above results indicate specific binding of affi_EGFR_-OMVs to the cells expressing EGFR receptors due to the presence of the Z_EGFR_ affibody.

### Affi_EGFR_-OMVs Bound to EGFR-Overexpressing MDA-MB-468 Cells

**FIGURE 4:**
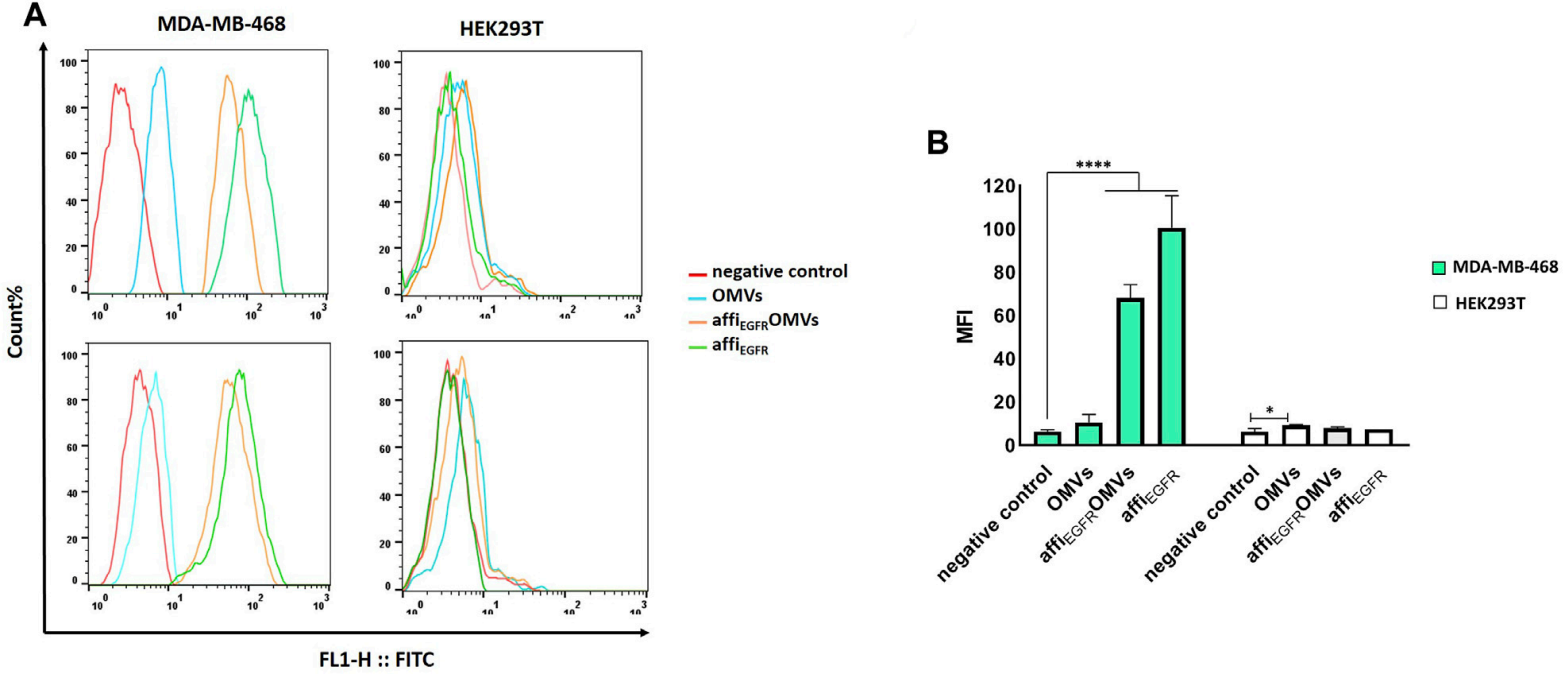
Flow cytometric analysis of affi_EGFR_-OMVs that bind to tumor cells. Analysis was carried out using OMVs and affi_EGFR_-OMVs binding to MDA-MB-468 and HEK293T cells. **(A)** The affi_EGFR_-OMVs and the purified affi_EGFR_ specifically attach to the EGFR-positive MDA-MB-468 cells as presented by shift in the fluorescence in MDA-MB-468 cells. **(B)** Plot of the MFI values in MDA-MB-468 and HEK293T cells incubated with the OMVs and the purified affi_EGFR_-GALA. Mean Fluorescence Intensity (MFI) value of negative control, i.e., incubation with anti-affibody and FITC-conjugated IgG alone, ruling out any unspecific binding to the tumor cells. 5 × 10^5^ numbers of cells per sample were recorded and MFIs are displayed as histograms. *p < 0.05, ****p < 0.0001 compared to negative control.

The binding property of the affi_EGFR_-OMVs was also compared to that of non-targeted OMVs in MDA-MB-468 and HEK293T cells using flow cytometry (Figures 4A,B). In parallel, affi_EGFR_ was utilized in this assay as a positive control. As illustrated in the results, the shift in fluorescence intensity for the affi_EGFR_-OMVs and affi_EGFR_ is well associated with the expression level of the receptor on the MDA-MB-468 cell surface; i.e, cells having a higher EGFR expression level revealed a greater shift than the non-EGFR-expressing HEK293T cell line. The non-targeted OMVs exhibited no binding to HEK293T and MDA-MB-468 cells ruling out any nonspecific binding of OMVs to the tumor cells under investigation. These results are in agreement with the ELISA data addressing the specific binding of the affi_EGFR_-OMVs to the EGF receptor.

**FIGURE 5:**
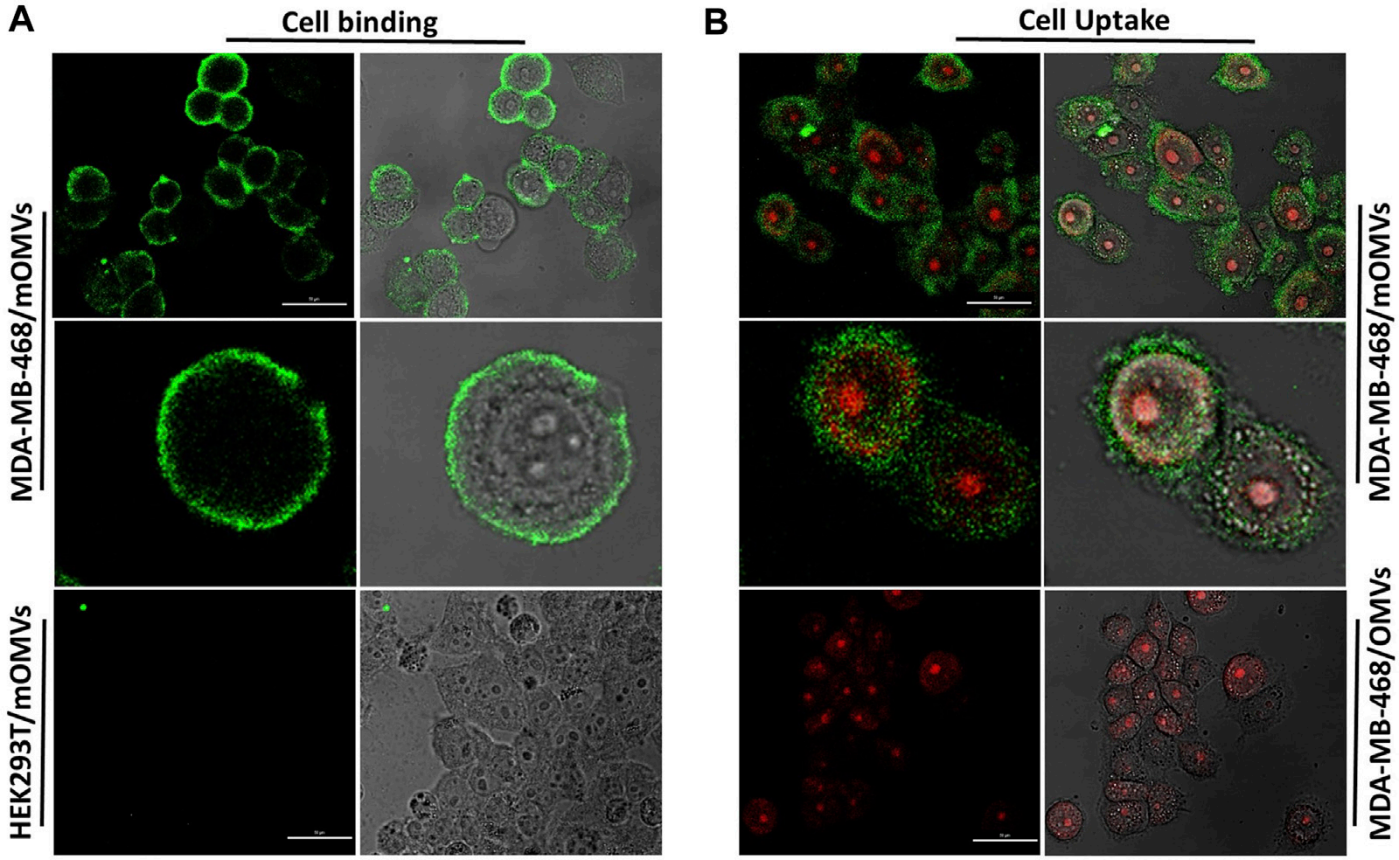
Cellular **(A)** binding and **(B)** uptake of the affi_EGFR_-OMVs analyzed by confocal microscopy. HEK293T and MDA-MB-468 cells were incubated with the affi_EGFR_-OMVs (modified OMVs: mOMVs) for 2 h at 4°C. No attachment was seen on HEK293T cells following incubation with the affi_EGFR_-OMVs. Affi_EGFR_-OMVs were internalized into the MDA-MB-468 cells after 2 h of incubation at 37°C, while no internalization was observed using OMVs into the MDA-MB468 cells. Cells were stained with anti-affibody antibody and FITC- conjugated rabbit anti-goat IgG. Scale bar in un-zoomed pictures represents 50 μm.

We also found no detection of affi_EGFR_-OMVs and affi_EGFR_ when EGFR-negative HEK293T cells were used since neither a fluorescent shift nor an enhancement in intensity was detected relative to the negative control under the same conditions. Interaction between the engineered OMVs and MDA-MB-468 cells was further investigated by confocal microscopy after co-culturing of the cells with 25 µg of the affi_EGFR_-OMVs for 2 h at 4°C. As it can be seen in Figure 5A, the affi_EGFR_-OMVs clearly bound to the surface of MDA-MB-468, whereas no binding was observed for HEK293T cells, used as the EGFR deficient cells. The findings of the confocal microscopy were in parallel with those of our previous assays, confirming that the affi_EGFR_-OMVs can selectively and effectively target EGF receptors.

### Internalization of the Affi_EGFR_-OMVs Into EGFR-Overexpressing MDA-MB-468 Cells

To further study the uptake of the OMVs by the cancer cells, MDA-MB-468 were treated with the non-targeted OMVs and affi_EGFR_-OMVs for 2 h at 37°C. Confocal microscopy revealed the cellular entry of the affi_EGFR_-OMVs, with red and green fluorescence pointing to the location of cell nuclei and OMVs, respectively, while no fluorescence was observed in the MDA-MB-468 cells treated with the non-targeted OMVs (Figure 5B).

### Affi_EGFR_-OMVs Compete With Epidermal Growth Factor and Cetuximab for Binding and Uptake by MDA-MB-468 Cells

**FIGURE 6:**
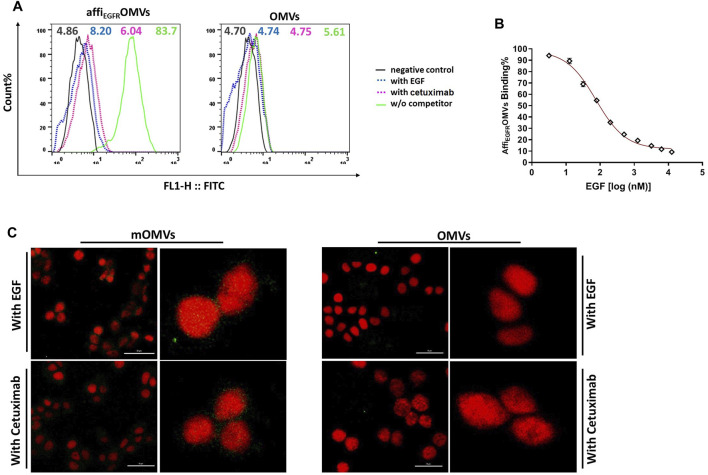
Competition binding and delivery analyses. **(A)** MDA-MB-468 cells were pretreated with competitors (EGF or Cetuximab) and then treated with the affi_EGFR_-OMVs. The flow cytometric results of cellular binding indicated that pretreatment of MDA-MB-468 with EGF or Cetuximab caused a significant shift in the fluorescence. **(B)** Affi_EGFR_-OMVs treated MDA-MB-4568 cells were co-incubated with the increasing amounts of EGF for 1 h. As declared by flow cytometric results, binding of the affi_EGFR_-OMVs to target cells gradually decreased in the presence of increasing amounts of EGF. **(C)** The MDA-MB-468 cells were pretreated with an excess amount of the competitors. The uptake data from confocal microscopy demonstrated a significant reduction in green fluorescence intensity of the modified OMVs-treated cells. Scale bar in un-zoomed pictures represents 50 μm. Cells were stained with anti-affibody antibody and FITC- conjugated rabbit anti-goat IgG.

To further investigate the specific binding of the affi_EGFR_-OMVs, EGFR-specific ligands, EGF, or Cetuximab was applied to compete with the affi_EGFR_-OMVs for binding to EGFR-overexpressing MDA-MB-468 cells. The flow cytometric data verified a specific interaction between affi_EGFR_-OMVs and MDA-MB-468 cells. As evidenced in Figure 6A, pretreatment with an excess amount of EGF or Cetuximab heavily blocked affi_EGFR_-OMVs binding to the target cells (8.20 and 6.04 MFI, respectively), while no significant fluorescent shift was observed in the case of OMVs. Moreover, co-incubation with 3.25 nM–12.5 μM of EGF resulted in a decrease of fluorescent intensity from 89.43 to 9.2% (Figure 6B). Indeed, these data indicate a gradual reduction in the binding ability of affi_EGFR_-OMVs to the receptors with increasing amounts of EGF, further supporting the specific binding to EGF receptors.

We also assessed the target-specific delivery of the affi_EGFR_-OMVs. To do it, EGF or Cetuximab pretreated MDA-MB-468 cells were examined with a fluorescence confocal microscope. As evidenced in the data, a significant reduction in green fluorescence intensity was visualized following the pretreatment of MDA-MB-468 cells with an excess amount of the competitors, EGF and Cetuximab (Figure 6C).

Overall, the competition results further demonstrated the target-specific affi_EGFR_-OMVs delivery to the cells.

### No Epidermal Growth Factor Receptor-1 Activation Following Affi_EGFR_-OMVs Treatment

**FIGURE 7:**
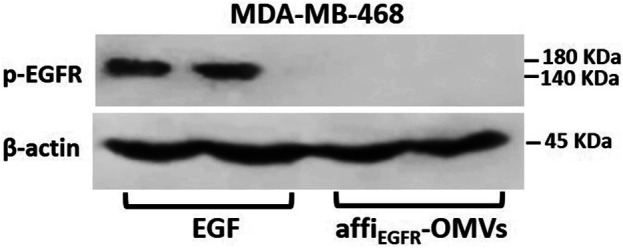
EGFR activation in MDA-MB-468 cells. Overnight starved MDA-MB-468 cells were treated with affi_EGFR_-OMVs (250 μg/ml) or EGF (100 nM) for 30 min at 37°C prior to lysis. Cell lysates were subjected to Western blotting analysis. Representative Western blot bands for p-EGFR and β-actin are shown along with two replicate bands for the affi_EGFR_-OMVs and EGF treatments. β-Actin bands are presented as a control of the loading amount of protein.

Autophosphorylation of EGFR as the primary transformation that happens upon ligand induction is a crucial characteristic of EGFR activation (Lemmon and Schlessinger, 2010). To examine if the affi_EGFR_-OMVs activate EGF receptors, phosphorylation of EGFR was monitored following treatment of MDA-MB-468 cells with affi_EGFR_-OMVs and EGF as a positive control. Western blotting results using EGFR phosphorylation site-specific antibody (Tyr1068) exhibited a significant band indicating expression of the phosphorylated EGFR in the EGF-treated samples. In contrast, no phosphorylated protein expression level was detected in the affi_EGFR_-OMVs treated cells (Figure 7). These findings confirmed that OMVs presenting affibody target cancer cells with no receptor stimulation effect.

### Safety Assessment of the Engineered Outer Membrane Vesicles

Besides the efficacy of targeting, the safety issue is a pivotal concern in the development of nanomedicine, where a bacterium-derived product is utilized. Since OMVs consist of a large portion of LPS activating toll-like receptors (TLRs), which in turn provokes an inflammatory response (Kuehn and Kesty, 2005), we thus evaluated OMV-induced inflammatory response by assessing TNF-α level and cytotoxicity *in vitro*. We also performed hemolysis and sterility studies and measured the endotoxin levels as well to assure the biocompatibility of the OMVs.

### The Engineered OMVs Showed No Endotoxicity

Findings from the gel clot LAL test showed the absence of a stable solid clot in the test tubes at the end of reactions confirming the lack of LPS for affi_EGFR_-OMVs and its lysate at all the concentrations used. The lack of endotoxin activity in the intact OMVs and the OMVs lysate, in which the lipid-A component of LPS is exposed, confirmed the exploitation of the LPS-modified ∆msbB/∆pagP W3110 strain in the current study.

### No Bacterial Growth Was Observed for the Engineered OMVs

**FIGURE 8:**
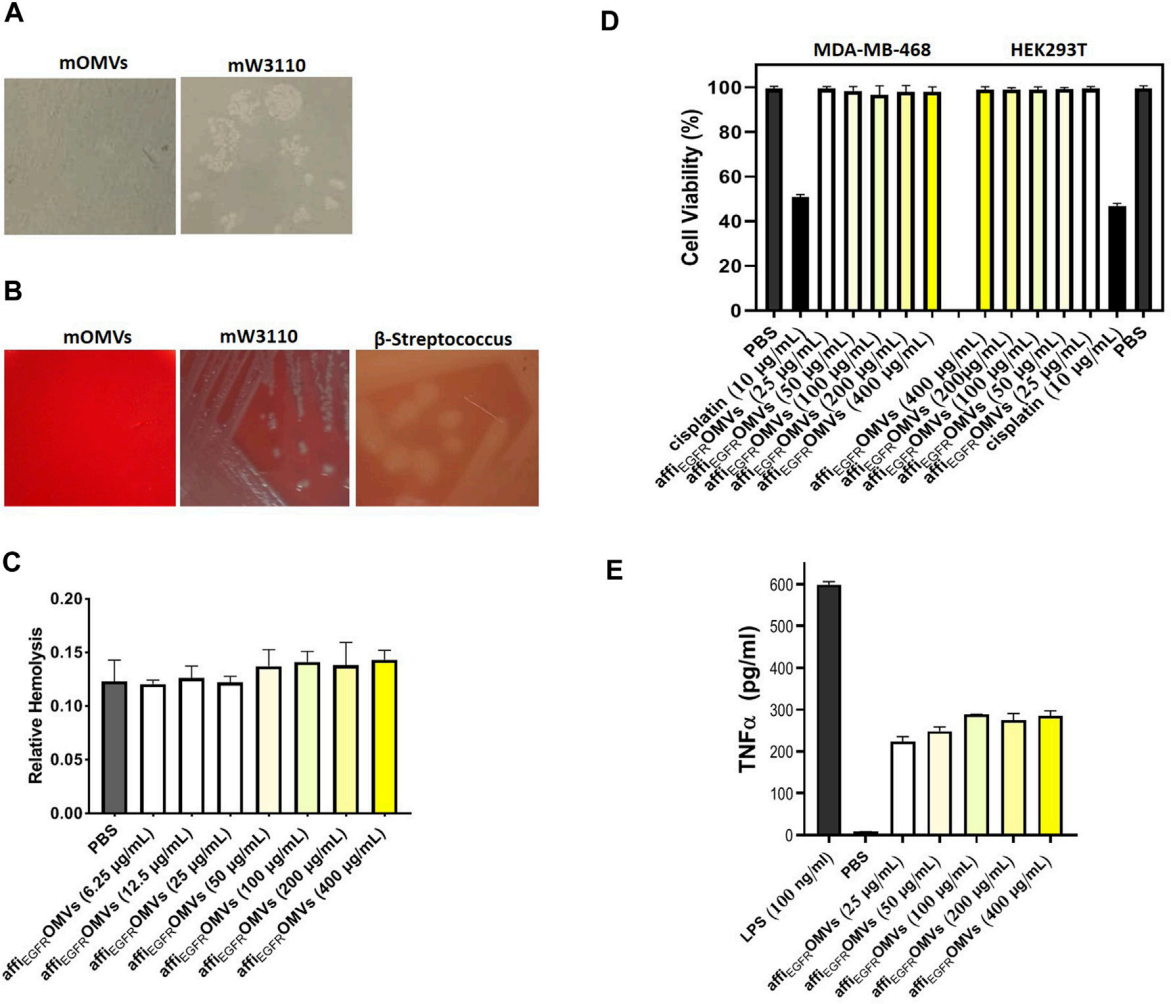
*In vitro* cytotoxicity, hemolytic activity, and immune stimulation assessments of the affi_EGFR_-OMVs. **(A)** No bacterial growth was seen on LB-agar plate cultured with the engineered OMVs (mOMVs), confirming sterility of the OMV sample. **(B)** Lack of hemolytic activity in affi_EGFR_-OMVs (mOMVs) by plating them on horse blood agar plates. A clear zone due to the red blood cell lysis was apparent in the β-*streptococcus* plate (positive control), whereas no lysis was observed in the bacteria and their derived affi_EGFR_-OMVs. **(C)** Liquide hemolytic activity test showed no erythrocyte lysis of the engineered OMVs. **(D)** Percentage of the cell viability in MDA-MB-468 and HEK293T cells, 72 h after treatment with affi_EGFR_-OMVs (mOMVs), no significant difference was observed in cell viability when treated with mOMVs and PBS as a negative control. Cisplatin (10 µg/ml) was used as a positive control. **(E)** TNF-α secretion from the THP1 cells was quantified by ELISA. The TNF-α level was significantly higher in the THP1 cells treated with LPS (100 ng/ml) than that of the cells treated with affi_EGFR_-OMVs.

The sterility of the purified OMVs was determined by plating the affi_EGFR-OMVs_ on LB-agar plates and incubating at 37°C overnight. The absence of the bacterial colonies proved that the OMV products were bacteria-free (Figure 8A).

### The Engineered OMVs Revealed No Hemolytic Activity

To validate the hemolytic activity of the affi_EGFR_-OMVs, the blood agar plated OMVs were prepared under the standard incubation conditions. No hemolysis was visualized upon plating the affi_EGFR_-OMVs and its origin bacteria, while clear zones were seen owing to the β-*streptococcus* strain culturing as a positive control (Figure 8B). The data from the quantitative liquid hemolytic assay supported our findings and displayed no considerable difference in erythrocyte hemolysis compared to PBS as the negative control (Figure 8C). These observations indicated that ClyA fusion protein is in an inert state and the affi_EGFR_-OMVs are safe enough to be used as a nanocarrier.

### The Engineered OMVs Had No Effect on the Cell Viability

Our data on the MTT assay on HEK293T and MDA-MB-468 cell lines revealed no significant alteration in terms of viability following transfection with the affi_EGFR_-OMVs compared to the PBS-treated cells, while cell viability was reduced to about 50% following Cisplatin treatment as a positive control. In other words, the normal appearance of the cells in both treatments implies no cytotoxic effect associated with the affi_EGFR_-OMVs (Figure 8D).

### No Immunogenicity for the Engineered OMVs

Based on a previous study reporting on the potent stimulatory activity for LPS in the production of TNF-α in THP1 cells, we next investigated the stimulation of the immune system by affi_EGFR_-OMVs in these cells and found that the affi_EGFR_-OMVs caused a slight increase in TNF-α level in human monocyte THP1 cells compared to cells induced with LPS (Figure 8E).

The desired findings from our *in vitro* assays pointed out the safety and biocompatibility of the engineered OMVs as a drug carrier in cancer research.

## Discussion

Targeted cancer therapy is one of the most cost-effective strategies to prevent severe and often long-lasting side effects of chemotherapy (Miler et al., 2016). Hence, the need for exploring and developing novel targeted nanoparticle delivery platforms remains the main subject to achieve more effective and reliable therapies. In this context, OMVs derived from genetically manipulated bacterial strains offer great potential for cancer targeting and are introduced as valuable drug carriers in cancer therapy (Gujrati et al., 2014). These particles are efficient in shielding their content and protect them against proteases, leading to an increased circulating half-life of the nanodrugs (Gujrati et al., 2014; Kim et al., 2017). Furthermore, by appropriate engineering, OMVs can target the specific cancer cells, which in turn reduce the unwanted side effects of the associated drugs (Kim et al., 2017). Given these data and considering that EGF receptors have been the subject of numerous cancer studies, the work presented here exploits the bacterial OMVs in targeting EGF receptors in triple-negative breast cancer cells. Triple-negative breast cancer (TNBC) has an aggressive feature, and so far, there is no available agent to target this type of tumor (Engebraaten et al., 2013; Cao et al., 2019; Damaskos et al., 2019). This motivated us to prepare the surface-modified OMVs by genetic fusion of Z_EGFR:_1907 affibody to the C-terminus of ClyA to increase the chance of presenting a properly folded affi_EGFR_ protein. As previous studies have demonstrated (Kim et al., 2008; Chen et al., 2010; Huang et al., 2016), the addition of ClyA as a leader sequence to the N-termini of the affibody assures the construct to be presented on the surface of the OMVs. This approach has been successfully employed in other studies using either OmpA or ClyA depending on the location of the proteins, i.e., into the lumen or on the surface of OMVs (Fantappie et al., 2014). In this line, we prepared the engineered OMVs for proof of concept of applying OMVs not only as an effectual antigen delivery system but also as a targeted nanocarrier.

Interestingly, the small size of the Z_EGFR:_1907 affibody (∼7 kDa) is an advantage to keep the size and morphology of OMVs unchanged. This was confirmed by DLS and TEM data. Apart from the small size of the Z_EGFR:_1907 affibody, the selectivity and high affinity to EGF receptors are the most important features of this molecule. Z_EGFR:_1907 is the second-generation of EGFR-specific affibody molecules, demonstrating a desirable tumor localization with no cross-binding to other growth factor receptors (Tolmachev et al., 2009). Previous studies have found that this affibody could be an appropriate candidate for targeted imaging in EGFR-expressing cells, including A431 and HCC tumors (Zhao et al., 2013). Consistently, our ELISA findings using EGFR protein revealed a satisfying affinity for both the purified Z_EGFR_ affibody-GALA (K_d_: 2.845 μg/ml) and Z_EGFR_ affibody presented on the surface of OMVs (K_d_: 86.41 μg/ml). From the K_d_ values, we speculated that the affi_EGFR_-OMVs contain about 3% of affibody-GALA out of the total OMV proteins, which surprisingly is similar to those estimated by Western blotting analysis (ranging from 1 to 5%) (Fantappie et al., 2014). Furthermore, this amount of affibody surface displacement was enough to effectively target MDA-MB-468 cells, as indicated by flow cytometry and ELISA analyses. From the results herein, we visualized that cell binding of the affi_EGFR_-OMVs was the same as the purified affibody-GALA, suggesting that OMVs display a full affibody-GALA protein with a proper conformation on their surface.

Considering that affi_EGFR_ interacts with a site on domain III of EGFR, which overlaps with the EGF binding site (Nordberg et al., 2007), we next covered the binding site with an excess amount of EGF or Cetuximab, then added the modified OMVs, and evaluated fluorescent intensity using flow cytometry. The results demonstrated that the nonspecific cellular binding is low for the modified OMVs, providing further insight into the EGFR receptor-mediated cellular interaction. Moreover, our findings revealed that binding capacity of the engineered-OMVs gradually reduced when increasing concentrations of EGF was added. These results were in agreement with our previous experiments in further confirming the targeted delivery system.

Since triple-negative breast cancer is known to overexpress EGFR and the EGF receptor is a well-established cancer therapy target (Seshacharyulu et al., 2012), using *E. coli* OMVs and the ClyA displaying system, we introduced a platform with a capability of specifically targeting triple-negative breast cancer, which can be further employed for cancers overexpressing EGF receptors. It is worth mentioning that several studies have proved that the expression of EGFR in triple-negative breast carcinoma is significantly higher than that of normal cells (Tang et al., 2012). However, the potential toxicity of the therapeutics targeting EGFR toward normal tissues is still challenging. This encouraged us to reveal the specificity of our construct toward the triple-negative breast cancer cells. Among a series of experiments conducted, immunofluorescence imaging of the MDA-MB-468 cells treated with the affi_EGFR_-OMVs presented an alternative approach to assure cell specificity. The data obtained indicated that the anti-EGFR engineered OMVs strongly bind to EGFR-overexpressing target cells, while no binding was detected when EGFR-negative cells were used, providing further evidence that this adherence was *via* interaction between Z_EGFR_ affibody and the EGF receptor. Interestingly, we also verified that no EGFR activation occurs following the adherence of the targeted OMVs to EGF receptors by measuring the level of phosphorylated EGFR expression. Furthermore, a critical concern when applying OMVs in cancer therapy is intracellular delivery. To this end, images taken by confocal microscopy confirmed the efficient uptake of the engineered OMVs by the EGFR-overexpressing cells. Conversely, the non-targeted OMVs were unable to enter the cancer cells. Since the nonspecific process can cause increased OMVs uptake, we also captured confocal images of the target cells pretreated with EGF or anti-EGFR mAb Cetuximab. The images validated the co-internalization of the modified OMVs with EGF receptors and exhibited that the modified OMVs competed with both Cetuximab and EGF in binding to EGFR and cellular uptake. These findings were in parallel with the report by Kim et al., indicating the incorporation of EGF on the surface of OMVs to efficiently bind to tumor cells expressing EGF receptors *in vitro* (Kim et al., 2017). However, a major concern in this study was the mitogenic and neoangiogenic properties of EGF. Recently, HER2 was selected as a target and anti-HER2 affibody expressed on the surface of OMVs was used for cancer-specific targeting due to its very high affinity and small size (Gujrati et al., 2014), which led us to design our engineered OMVs toward triple-negative breast cancer.

Of note, one of the major barriers in the cytosolic delivery of biomacromolecules is endocytosis; thus, efficiency to escape endosome seems to be important (Kakudo et al., 2004; Varkouhi et al., 2011; Burks et al., 2015; Niikura et al., 2015). To make sure of endosomal escape, GALA was fused to the C-terminus of the His tag-Z_EGFR_ fusion protein. Consistent with our study, a recent trial using a confocal laser scanning microscope (CLSM) showed the endosomal scape capability of a Z_HER2_ affibody displaying bio-nanocapsule following fusing with GALA on the surface of the particle (Nishimura et al., 2014). Indeed, we focused our attention on representing a potential model for a delivery system by genetically engineering OMVs equipped with GALA in which multiple parts would act in concert to accomplish an effectual and specific delivery platform to be used in EGFR-positive breast cancer therapy.

On the other hand, a key concern with the use of OMVs is the presence of LPS (Ellis et al., 2010; Vanaja et al., 2016). Since OMVs are released from the outer membrane of bacteria, they have intrinsic inflammatory potential owing to their LPS content (Kuehn and Kesty, 2005). In other words, an integral pro-inflammatory component of LPS, the hexa-acylated lipid-A, stimulates the immune system through activating toll-like receptors pathways (Ellis et al., 2010). Here, we used an *E. coli* W3110 strain carrying double mutations of the msbB and pagP genes, which is able to generate OMVs with only penta-acylated LPS. The endotoxic activity of the modified OMVs from this double mutant has been attenuated several times compared to the wild-type W3110 (Lee et al., 2011). Hence, our engineered OMVs are supposed to stimulate a minimum immunogenic and toxic response, avoiding the possible undesirable or even fatal effects during the treatment process (Gujrati et al., 2014; Alves et al., 2015).

Of interest, our *in vitro* immune-stimulating assessment revealed no significant increase in the level of TNFα even at high concentrations in THP1 cell culture media following incubation with the affi_EGFR_-OMVs compared to LPS as a positive control. For further verification, the hemolytic activity of the engineered OMVs was compared to that of the original bacteria using blood agar plates, and the liquid hemolysis assay was simultaneously performed as well. The results indicated that ClyA-Z_EGFR_ GALA fusion protein is non-hemolytic. Further support for our results is that ClyA (SheA), a protein known for its hemolytic activity, has a latent/silenced structural gene in *E. coli* K-12 so that under many tested laboratory conditions, it is phenotypically silent in *E. coli* K-12 (Oscarsson et al., 1999; Westermark et al., 2000; Kim et al., 2008). Interestingly, it was reported that the absence of functional ClyA expression in an *E. coli* host can facilitate the translocation of the recombinant ClyA-fusions to the outer membrane and the presentation of fusion partner on the derived OMVs (Huang et al., 2016). In this study, surface localization of the Z_EGFR_ GALA using ClyA as a leader protein was confirmed by immune fluorescence staining and flow cytometry.

Moreover, targeted OMVs are needed to rule out toxic side effects. Cell viability findings displayed no significant difference between cells incubated with the affi_EGFR_-OMVs and PBS, confirming the biosafety of the engineered OMVs for our future design and development of OMVs-based studies to develop effective and well-tolerated chemotherapeutics.

Naturally occurring vesicles such as OMVs provide a number of therapeutic advantages, as addressed earlier (Gu et al., 2020); however, no clinical studies have yet identified OMVs as a drug delivery vehicle. Indeed, despite developments in the employment of the extracellular vesicles in clinical trials, many challenges are needed to be overcome prior to a successful therapeutic application. As a consequence of the aforementioned caveats, the trials are limited to a few cases. In this context, phase I clinical trials have been performed using tumor peptide/drug–loaded exosomes in patients with metastatic melanoma, advanced non-small cell lung cancer (NSCLC), colorectal cancer (CRC), and malignant glioma (Thomas Jefferson University Hospital, 2000; Escudier et al., 2005; Morse et al., 2005; Dai et al., 2008; Ohno et al., 2016). Moreover, two phases I of clinical trials have recently assessed the oral plexosome-based curcumin delivery to normal and cancerous colon tissues (James Graham Brown Cancer Center, 2000). In the case of OMVs, phase I and II clinical trials only demonstrated the protective effect of OMV-based vaccine against *N. meningitidis* (de Kleijn et al., 2000; Marsay et al., 2015; Zhang et al., 2019).

The findings of this study are the first step toward achieving a targeted drug delivery system, which paves the way for further investigation on verification in the relevant *in vivo* and clinical models.

## Conclusion

In the light of the foregoing results, we concluded that the EGFR affibody can be directed onto the OMV surface *via* ClyA fusion. The success of targeting triple-negative breast cancer cells by the affi_EGFR_-OMVs followed by the internalization of the vesicles with no cytotoxic, hemolytic, or immune-activating effects is an interesting finding. However, research concerning the application of OMVs in cancer therapy is just in its infancy.

## Data Availability

The original contributions presented in the study are included in the article/Supplementary Material; further inquiries can be directed to the corresponding author.

## Abbreviations

BSA, Bovine albumin serum; ClyA, Cytolysin-A; DLS, Dynamic Light Scattering; DMED, Dulbecco’s Modified Eagle’s Medium; *E. coli*, Escherichia coli; EGF, Epidermal growth factor; EGFRs, Epidermal growth factor receptors; ELISA, Enzyme-linked immunosorbent assay; FBS, Fetal bovine serum; HRP, Horseradish peroxidase; IPTG, Isopropyl β-d-1-thiogalactopyranoside; LAL, Limulus amoebocyte lysate; LB, Luria–Bertani LB; LPS, Lipopolysaccharide; OMVs, Outer membrane vesicles; mOMVs, Modified OMVs, PBS: Phosphate-buffered saline; PDI, Polydispersity index; PFA, Paraformaldehyde; PMA, Phorbol myristate acetate; RPMI, Roswell Park Memorial Institute; SDS-PAGE, Sodium dodecyl sulfate-polyacrylamide Gel; TEM, Transmission electron microscopy; TLR, Toll-like receptors; TNBC, Triple-negative breast cancer.
