# Supplementary figures and images for: Surface Engineering of Escherichia coli–Derived OMVs as Promising Nano-Carriers to Target EGFR-Overexpressing Breast Cancer Cells
Source: Front Pharmacol. 2021 Nov 18;12:719289. doi: 10.3389/fphar.2021.719289 (PMC8638777; doi:10.3389/fphar.2021.719289)

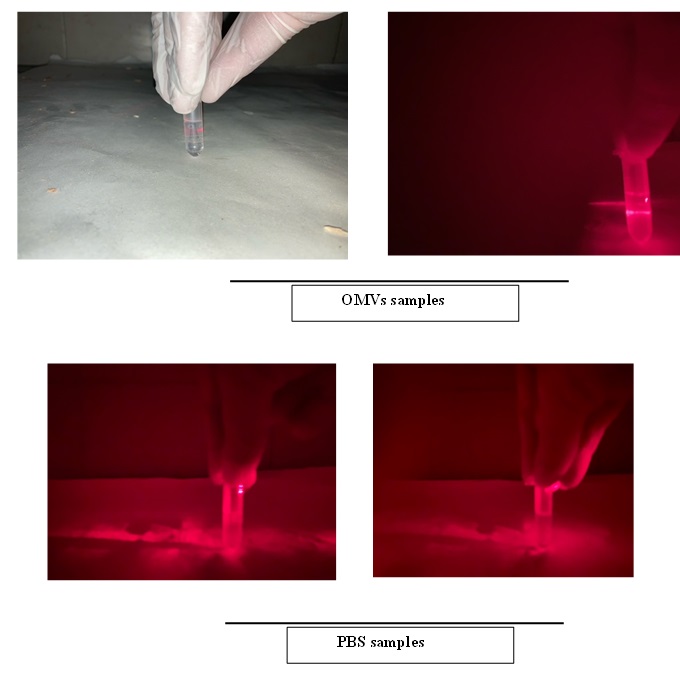

Supplement: Supplementary file 1 [file Image1.JPEG]

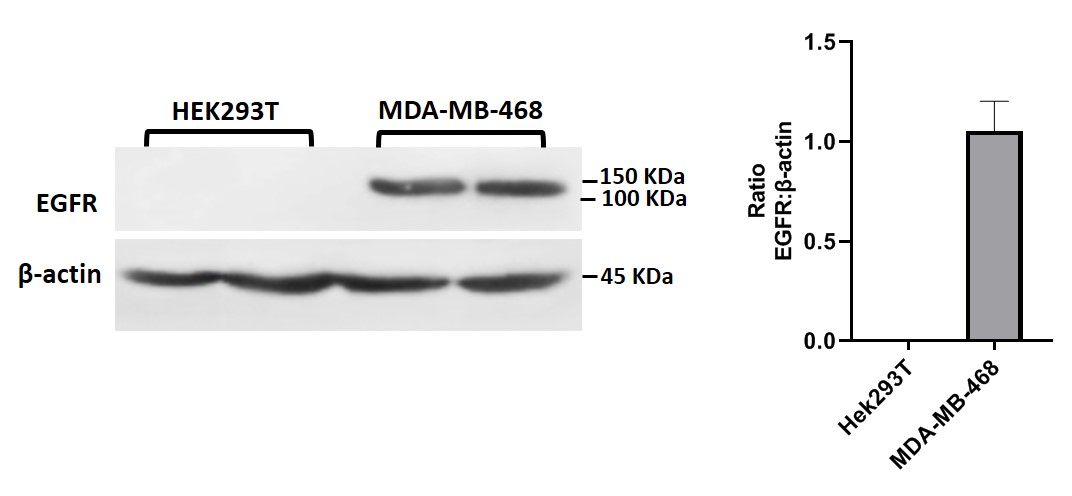

Supplement: Supplementary file 2 [file Image2.JPEG]
